# Lessons from Genetic Studies of Primary Immunodeficiencies in a Highly Consanguineous Population

**DOI:** 10.3389/fimmu.2017.00737

**Published:** 2017-06-27

**Authors:** Mohamed-Ridha Barbouche, Najla Mekki, Meriem Ben-Ali, Imen Ben-Mustapha

**Affiliations:** ^1^Laboratory of Transmission, Control and Immunobiology of Infection (LR11IPT02), Institut Pasteur de Tunis, Tunis, Tunisia; ^2^Faculty of Medicine, Université de Tunis El Manar, Tunis, Tunisia

**Keywords:** primary immunodeficiencies, consanguinity, autosomal recessive, founder effect, genetic counseling

## Abstract

During the last decades, the study of primary immunodeficiencies (PIDs) has contributed tremendously to unravel novel pathways involved in a variety of immune responses. Many of these PIDs have an autosomal recessive (AR) mode of inheritance. Thus, the investigation of the molecular basis of PIDs is particularly relevant in consanguineous populations from Middle East and North Africa (MENA). Although significant efforts have been made in recent years to develop genetic testing across the MENA region, few comprehensive studies reporting molecular basis of PIDs in these settings are available. Herein, we review genetic characteristics of PIDs identified in 168 patients from an inbred Tunisian population. A spectrum of 25 genes involved was analyzed. We show that AR forms compared to X-linked or autosomal dominant forms are clearly the most frequent. Furthermore, the study of informative consanguineous families did allow the identification of a novel hyper-IgE syndrome linked to phosphoglucomutase 3 mutations. We did also report a novel form of autoimmune lymphoproliferative syndrome caused by homozygous FAS mutations with normal or residual protein expression as well as a novel AR transcription factor 3 deficiency. Finally, we identified several founder effects for specific AR mutations. This did facilitate the implementation of preventive approaches through genetic counseling in affected consanguineous families. All together, these findings highlight the specific nature of highly consanguineous populations and confirm the importance of unraveling the molecular basis of genetic diseases in this context. Besides providing a better fundamental knowledge of novel pathways, their study is improving diagnosis strategies and appropriate care.

## Introduction

The expression of recessive genes inherited from a common ancestor, in consanguineous populations, underlies higher frequency of otherwise rare genetic diseases (1–3). Indeed, the molecular basis of many monogenic disorders has been first characterized in highly inbred populations. Furthermore, in a significant proportion of cases, a strong founder effect for specific mutations has been reported in these settings; some mutations might be unique to a particular endogamous community (4, 5).

Primary immunodeficiencies (PIDs) are a heterogeneous group of genetic disorders of the immune system that predispose patients to infections, autoimmune diseases, lymphoproliferation, and malignancy. The exact prevalence of PIDs worldwide is unknown but is expected to be more common in areas with high rates of consanguinity (6). The autosomal recessive (AR) forms of these disorders represent the most frequent mode of inheritance as compared to X-linked (XL) or autosomal dominant (AD) forms. Indeed, according to the 2015 IUIS classification of PIDs, 206 out of 289 known forms of PIDs are AR (7). The occurrence of the disease in the progeny of unaffected consanguineous parents is highly suggestive of an AR mode of inheritance. However, molecular studies are required to confirm the AR determinism of disease in these PIDs affected families.

Although significant efforts have been made in recent years to develop genetic testing across the Middle East and North Africa (MENA) region, very few comprehensive studies reporting molecular basis of PIDs in these settings are available. Herein, we review genetic characteristics of PIDs identified in a large series of inbred Tunisian (North-African) patients. We outline the molecular basis of disease in this population and its potential contribution to a better care through genetic counseling. Furthermore, we discuss the relevance of such studies in the discovery of new PIDs genes and novel modes of inheritance for known PIDs.

## PIDs Distribution, Parental Consanguinity, and Familial History

This review analyzes data collected from different genetic studies performed in a total of 168 PIDs patients belonging to 122 kindreds. This is at the best of our knowledge, one of the largest molecular studies of PIDs patients from highly consanguineous MENA populations along with the study from Saudi Arabia (8). The most frequently observed PIDs in this series include combined T and B cell immunodeficiencies and congenital defects of phagocyte that account for 52 (30.95%) and 37 (22%) patients, respectively, contrasting with data from European series showing the predominance of antibody deficiencies (9). Indeed, only 15.47% of the patients had predominantly antibody deficiencies. Similar observations have been reported in others series from MENA region (10, 11). This could be due to the less severe clinical phenotype and to the lack of adult physicians’ awareness particularly with regard to common variable immunodeficiency. Moreover, the high frequency of particular AR forms of combined immunodeficiencies among North-African PIDs patients could also account for such findings. Indeed, major histocompatibility complex (MHC) class II deficiency is the most frequently reported PID entity in this series with 27 patients, and the majority of patients reported worldwide are of North-African origin (Algeria, Tunisia, and Morocco) (12). Other categories of PIDs included defects in intrinsic and innate immunity (23 cases), combined immunodeficiencies with associated or syndromic features (19 cases), and diseases of immune dysregulation (11 cases). Furthermore, few patients presenting rare PIDs with peculiar clinical and/or immunological phenotype did also undergo genetic characterization. Altogether, the pattern of PIDs distribution in patients with established molecular diagnosis in this series is representative of the actual distribution of PIDs reported by the few national registries available from MENA region (13).

Interestingly, Tunisian and other MENA region countries are unique with regard to high prevalence of consanguinity in the general population varying between 20 and 50% of all marriages (13). Consistently, the patients studied in this series show a high rate of parental consanguinity that reaches 61.9% accounting for the high frequency of family history (55.35%) including early deaths, similar clinical features, and/or previously identified PIDs in relatives. Consanguinity rate was particularly high in patients with Omenn syndrome (88.8%), phosphoglucomutase 3 (PGM3) deficiency (85.7%), leukocyte adhesion deficiency type 1 (LAD I) (76.4%), and MHC class II deficiency (70.3%) (Table 1). This is in accordance with the results obtained for one of the largest series of MHC class II North-African patients reporting a consanguinity rate of 81.8% (12), as well as for LAD I and Omenn syndrome in other patients series originating from MENA region (14–16).

**Table 1:** Mode of inheritance and molecular studies in the 170 Tunisian patients investigated.

PID	Gene	Mode of inheritance	Number of kindred	Number of patients	Confirmed parental consanguinity	Molecular defects		Recurrent mutation	Novel mutation
						cDNA	Protein		

Immunodeficiencies affecting cellular and humoral immunity (*n* = 53)									
SCID	*IL2RG*	XL	1	2	–	c.865C>T	R289X	–	–
			1	3	–	c.710G>A	W237X	–	+
			1	2	–	c.222G>A	W74X	–	+
	*RAG 2*	AR	1	1	1	c.1219G>T	E407X	–	–
			2	2	2	c.1338C>G	C446W	–	–
	*IL7RA*	AR	1	1	1	c.616 C>T	R206X	–	–
	*PNP*	AR	1	1	1	c.181 + 1G>A	–	–	+
Omenn syndrome	*RAG1*	AR	7	9	8	631delT	T173TfsX28	+	–
MHC-II deficiency	*RFXANK*	AR	23	27	19	c.338-25_338del26	I5E6-25_I5E6 + 1	+	–
HIGE—DOCK8 deficiency	*DOCK8*	AR	1	1	1	Ex1-43 del	–	–	+
HIGM—CD40 ligand deficiency	*CD40LG*	XL	1	1	–	c.348_351dup	Q118Vfs*5	–	+
			1	1	–	c.782_*2del	L261Qfs*50	–	+
			1	1	–	c.[356G>A; 299_356del]	p.([116G>S, D97_K115del])	–	–
HIGM—CD40 deficiency	*CD40*	AR	1	1	1	c.109T>G	C37G	–	+

**Combined immunodeficiencies with associated or syndromic features (*n* = 19)**									

HIES	*STAT 3*	AD	1	4	–	c.1298A>G	M329V	–	+
			1	1	–	c.1858A>G	T620A	–	–
	*PGM3*	AR	3	12	10	c.1018_1020del	E340del	+	+
			1	2	2	c.248T>C	L83S	–	+

**Predominantly antibody deficiencies (*n* = 26)**									

XLA	*BTK*	XL	2	2	–	c.1762T>G	W588G	–	+
			1	1	1	c.863G>A	R288Q	–	–
			2	2	1	c.435C>A	C145X	–	–
			1	1	–	c.1117C>T	L373V	–	+
			1	1	–	c.1567 − 1G>A	–	–	+
			1	1	1	c.1181C>G	S394X	–	+
			1	2	2	c.1631 + 1G>A	–	–	–
			1	1	1	c.653delA	K218fsX228	–	–
			1	1	–	c.1845_1846insGT	L616fsX649	–	+
	*IGHM*	AR	1	1	1	c.1789insCC1792-1796del CCAGC	V378AfsX1	–	+
	*TCF3*	AR	1	2	2	c.808C>T	Q270X	–	+
HIGM—AID deficiency	*AICDA*	AR	1	1	–	c.91T>C	Y31H	–	+
			4	5	4	c.389A>C	H130P	+	+
			3	5	3	c.156 + 1G>T	([N53Lfs*15, N53Lfs*19])	+	–^a^

**Diseases of immune dysregulation (*n* = 10)**									

ALPS	*FAS*	AD	1	1	–	c.266G>A	A16T	–	–
			1	1	–	c.926G>A	E194K	–	–
			1	1	–	c.365C>T	T122I	–	–
			1	1	–	c.1009A>G	E256G	–	–
		AR	1	4	3	c.1017A>G	N266S	–	+
			1	2	1	c.581C>T	R121W	–	+

**Congenital defects of phagocyte number, function, or both (*n* = 37)**									

CGD	*NCF2*	AR	6	11	9	c.257 + 2T>C	A59IfsX2	+	–^a^
			1	1	1	c.78A>T	N419I	–	+
	*NCF1*	AR	5	5	2	c.75_76delGT	–	–	–
	*CYBA*	AR	1	1	–	295-301delGTGCCCG	–	–	+
			1	1	–	c.70G>A	G24R	–	–
	*CYBB*	XL	1	1	–	c.1359G>A	W453X	–	+
LAD I	*CD18*	AR	10	15	11	c.119_128delGGCCCGGCTG	G40A fsX7	+	–
			2	2	2	c.1777C>T	R593C	–	–

**Defects in intrinsic and innate immunity (*n* = 23)**									

MSMD	*IL12B*	AR	7	9	4	c.298_305del	–	+	+
	*IL12RB1*	AR	1	1	–	622C>A	C185X	–	+
			1	1	1	64 + 5G → A	–	–	+
			2	2	2	64 + 2T>G	–	–	–
			1	2	2	1386-1387delGT	–	–	+
			1	1	1	550-2A>G	–	–	+
			2	2	–	1791 + 2T>G	–	–	–
	*IFNGR1*	AR	2	3	2	c.131delC	–	+	–^a^
CMC	*STAT1* (GOF)	AD	1	1	1	c.876T4G	L163R	–	+
			1	1	–	c.820C>T	R274W	–	–

*^a^These mutations have been already reported for the first time in other patients from Tunisian origin*.

*XL, X-linked inheritance; AR, autosomal recessive inheritance; AD, autosomal dominant inheritance; SCID, severe combined immunodeficiencies; ALPS, autoimmune lymphoproliferative syndrome; AID, activation-induced cytidine deaminase; CGD, chronic granulomatous disease; MSMD, Mendelian susceptibility to mycobacterial disease; LAD, leukocyte adhesion deficiency; CMC, chronic mucocutaneous candidiasis; PID, primary immunodeficiency; MHC, major histocompatibility complex; LAD I, leukocyte adhesion deficiency type 1; HIGM, hyper-IgM syndrome; HIES, hyper-IgE syndrome*.

## Molecular Studies and Mode of Inheritance

In total, mutational studies of 25 candidate genes identified 58 different mutations as detailed in Table 1. Among them, 30 were novel (53.57%) with no records in three major databases including HGMD (The Human Gene Mutation Database), LOVD (Leiden Open Variation Database), and IDbases (databases for immunodeficiency-causing variations). Three mutations have been already reported for the first time in other patients from Tunisian origin (17–19) and nine mutations were recurrent. The identified mutations include 21 missense mutations, 10 nonsense mutations, 10 splice-site mutations, 12 deletions, 1 duplication, and 1 insertion. Interesting and rare mutational mechanisms included one complex mutation (2 pb insertion and 5 bp deletion) in *IGHM* gene (20), and one *de novo STAT1* mutation was identified in AD chronic mucocutaneous candidiasis disease (21). Mutations generating a premature stop codon were frequently observed in severe combined immunodeficiencies patients (71%), particularly those with *IL2RG* gene defect. All novel missense mutations were predicted to be possibly or probably damaging by Polyphen2 and/or SIFT algorithms. Furthermore, a deleterious effect was confirmed by appropriate functional testing for several gene mutations including *TCF3* (22), *AICDA* (23), *NCF2* (24), *FAS* (25), *STAT1* (21), *IL12B* (26), and *PGM3* (27).

These molecular studies confirm that the AR mode of inheritance is the most common in Tunisian patients accounting for 73% of all PIDs entities investigated. XL and AD modes were identified in only four and three different disorders, respectively. The deeply rooted tradition of parental consanguinity in the Tunisian general population, which remained relatively constant during the last four decades (28), has resulted in an elevated burden of AR PIDs since consanguinity favors the expression of recessive alleles (29). This is the case for two otherwise rare AR PIDs, namely, MHC class II deficiency and LAD I, diagnosed in fewer than 200 and 300 patients worldwide (12, 30) but accounting for 56 and 30 patients in Tunisia, respectively (31, 32). One practical implication related to this high frequency is to recommend, at least for the MHC class II deficiency, routine investigation of DR expression for North-African patients presenting symptoms suggestive of combined deficiency. Furthermore, for PIDs with more than one known mode of transmission, the AR trait was the most frequent or proportionately more represented in our settings than in other series and registries from non-consanguineous populations. Indeed, this mode of transmission accounted for most kindreds with chronic granulomatous disease (CGD) (93%), hyper-IgM syndrome (HIGM) (75%), and hyper-IgE syndrome (HIES) (71.42%). Consistently, among CGD patients, only one had a mutation in *CYBB* gene whereas the remaining patients (19/20) bear mutations in AR CGD genes. This is in accordance with previous reports showing a higher frequency of AR CGD in consanguineous populations (13, 33). In contrast, the XL form caused by *CYBB* gene mutation is the most common CGD form accounting for 70% of all patients according to the European registry data (34). Similar findings have also been observed in Tunisian patients with HIGM. Indeed, we demonstrate that 11 out of 15 patients are assigned to the AR form of the disease due to mutations in *AICDA* gene (20, 23). In contrast, the XL CD40L deficiency defines the most frequent type of HIGM in other series as it has been reported in 75% in North American and Asian patients (35), 42% in European patients (9), and 94% in Latin American patients (36). Thus, AR and XL forms for these diseases should be equally suspected in males originating from consanguineous regions. For HIES, PGM3 homozygous mutations account for 70% of Tunisian patients whereas the AD form due to mutations in signal transducer and activator of transcription 3 is the most common form reported worldwide (37).

## Identification of Novel Gene and of Novel Mode of Inheritance for Known Genes

Because a majority of PIDs are inherited as AR traits, the identification of patients with singular clinical and immunological phenotype within informative consanguineous families has tremendously contributed to the discovery of novel disease-causing genes. Accordingly, we did recently identify *PGM3* gene defect due to homozygous hypomorphic mutations in patients from two Tunisian consanguineous families with hyper-IgE like syndrome (27). This gene has not been previously associated with human disease. The patients’ clinical phenotype included classical features of HIES; however, they showed neurologic impairment with a developmental delay and psychomotor retardation (27). PGM3 enzyme plays an important role in the glycosylation pathway by catalyzing a key step in the synthesis of UDP-GlcNAc required for the biosynthesis of N-glycans (38). Thus, leukocytes from PGM3 deficient patients showed aberrant pattern of glycosylation due to altered PGM3 enzymatic activity (Table 2). This new congenital disorder of glycosylation accounts probably for the patients’ clinical and immunological phenotype, although the underlying mechanisms remain to be fully understood. Concurrently, additional patients with heterogeneous clinical phenotypes carrying distinct *PGM3* mutations have also been described (39, 40). Given that some congenital disorders of glycosylation are treatable with supplements of enzyme substrates (41), such an approach in these patients with hypomorphic mutations and residual enzymatic activity could be proposed to improve their condition while waiting the generally difficult access to bone marrow transplantation in our settings. Many other gene discoveries have been previously made following the study of large consanguineous families from the MENA region. Indeed, as already reported by Barbouche and Eley (42), during the period 1994–2013, at least 21 novel underlying genes were first described in PIDs families living or originating from this region.

**Table 2:** Identification of novel gene and novel mode of inheritance for known genes in Tunisian consanguineous families.

Identification strategy		Mutation	Protein expression	Functional consequences	Reference

Novel gene					
*PGM3*	Homozygosity mapping/linkage analysis and selector-based sequencing	c.1018_1020del (Hypomorphic)	Reduced	Reduced PGM3 enzymatic activity	(27)
		c.248T>C (Hypomorphic)			

**Novel mode of inheritance**					

*FAS*	Sanger sequencing	c.1017A>G (Loss of function)	Present	Resistance to Fas-mediated cell death	(25)
		c.581C>T (Loss of function)			
*TCF3*	Whole exome sequencing	c.808C>T (Loss of function)	Absent	Hypogammaglobulinemia	(22)

Families from areas with a high rate of consanguinity are important not only for the discovery of novel disease-causing genes but also for the identification of novel forms of known PIDs. Indeed, in autoimmune lymphoproliferative syndrome (ALPS), heterozygous germline mutations in the *FAS* gene inherited in an AD mode and associated with preserved protein expression are the most common cause of ALPS (25). These heterozygous mutations reported mainly in outbred human populations alter protein functioning by dominant-negative effect or by haploinsufficiency mechanisms (43–45). Very rare cases of total absence of Fas protein expression caused by homozygous *FAS* mutations have been reported (46). Interestingly, we identified in two unrelated consanguineous Tunisian kindreds the first example of a human AR ALPS characterized by homozygous *FAS* gene mutations in either intracellular or extracellular domains associated with normal or residual Fas expression, respectively. Both mutations are associated with resistance to Fas-mediated cell-death (25) (Table 2).

In addition, we identified another mode of inheritance for the previously reported AD agammaglobulinemia due to heterozygous dominant-negative *de novo* mutation in transcription factor 3 (*TCF3*) gene (47). Indeed, we did recently report a patient with a homozygous nonsense mutation in *TCF3* gene, who presented with severe hypogammaglobulinemia, very low number of B cells and developed B-cell acute lymphoblastic leukemia (22) (Table 2).

Both novel modes of inheritance herein reported are supported by parents’ consanguinity, familial history, and segregation of clinical and immunological features with the homozygous status. Such findings expand the spectrum of ALPS and TCF3 deficiency types and should prompt clinicians to search for such patients in highly endogamous populations for appropriate clinical and immunological follow-up.

## Investigations of Homozygosity Mapping and Age Estimation of Founder Mutations

Another striking feature pinpointed in the study population is the high frequency of founder effects accounting for the recurrence of mutations, which were unique for several PIDs. The patterns of founder mutation distribution disclosed two types: those that are shared with other populations particularly from the North-African countries and those that are specific to Tunisia. One of the most illustrative PIDs examples for a regional founder effect is the AR MHC class II combined immunodeficiency that has been considered to be a “North-African disease.” Indeed, we have identified a founder effect for the highly frequent c.338-25_338del26 mutation (also known as 752delG-25) in the RFXANK gene resulting in a 26-bp deletion (31) that has been reported in other North-African studies (4, 5, 48). The founder event responsible for this mutation has been estimated to have arise approximately 2,250 years ago (12), a period concurrent to the Berber civilization. This is consistent with previous reports showing that the population of the North Africa particularly in Tunisia, Algeria, and Morocco has a common substantial genetic background and that founder mutations could be shared in some of these countries (49). Additional MHC class II mutations have been since reported in the Middle East and other world regions (50–52). Another recurrent mutation in *RAG1* gene (631delT) has been identified in nine Tunisian patients with Omenn syndrome; its description was limited to patients originating from North Africa as well (53–56) suggesting a possible founder effect for this variant. Furthermore, a recent study reported the recurrence of the same homozygous mutation (Q289X) in *CARD9* gene in eight Algerian and four Tunisian patients from seven unrelated families with deep dermatophytosis (57). Such finding was due to a founder effect with the common ancestor living approximately 975 years ago (57).

Interestingly, other identified founder effects seem to be limited to Tunisian regions (49). Indeed, a founder effect for c.298_305del mutation in the *IL12B* gene has been reported in patients originating from the same Tunisian village. This mutation resulting in Mendelian susceptibility to mycobacterial disease is inherited as a common founder mutation arousing 1,100 years ago (26). In addition, we have recently reported a recurrent homozygous mutation in *NCF2* (c.257 + 2T>C) gene in 11 Tunisian patients CGD (24). The founder mutational event responsible of the recurrence of this mutation seems to be more recent since it was estimated to have occurred approximately 175 years ago (24).

Deleterious founder mutations have been reported in consanguineous populations to be the underlying cause of a large spectrum of monogenic AR diseases (28). In Tunisia, more than 300 genetic disorders were reported. Among them, 42% were associated to the presence of potential founder effect including PIDs (49, 58). Several clinical implications of the existence of the founder effect are the implementation of preventive approaches through genetic counseling and prenatal diagnosis in affected families. Furthermore, early genetic diagnosis in patients originating from the same geographical area will help propose HSCT, which is often the unique curative treatment, prior to the development of a severe clinical phenotype.

## Conclusion

This review shows evidence that AR forms of PIDs are frequent in our settings characterized by a high rate of consanguineous marriages. The presence of recurrent mutations and strong founder effects are shown to be common characteristics of PIDs patients from this population. Furthermore, these findings clearly demonstrate that the presence of an extended genetic homozygosity has the potential to reveal unusual patterns of inheritance. Thus, classical dominant disorders may assume a recessive pattern of inheritance in such population. Finally, it becomes obvious that PIDs in this region of the world is a relatively important health priority. The role of accurate molecular diagnosis and appropriate genetic counseling will help moderate the burden of PIDs and its associated costs for the community.

## Author Contributions

M-RB, NM, MB-A, and IB-M did contribute to the collection, analysis, and interpretation of the data as well as editing of the manuscript and approval of the submitted version.

## Conflict of Interest Statement

The authors declare that the research was conducted in the absence of any commercial or financial relationships that could be construed as a potential conflict of interest.
